# Microstructural analysis and calcite piezometry on hydrothermal veins: Insights into the deformation history of the Cocos Plate at Site U1414 (IODP Expedition 344)

**DOI:** 10.1002/2017TC004490

**Published:** 2017-08-18

**Authors:** Jennifer Brandstätter, Walter Kurz, Anna Rogowitz

**Affiliations:** ^1^ Institute of Earth Sciences, NAWI Graz Geocenter University of Graz Graz Austria; ^2^ Department for Geodynamics and Sedimentology University of Vienna Vienna Austria

**Keywords:** microstructures, calcite twins, veins, Cocos Plate, IODP Expedition 344, Site U1414

## Abstract

In this study we present microstructural data from hydrothermal veins in the sedimentary cover and the igneous basement recovered from Hole U1414A, Integrated Ocean Drilling Program (IODP) Expedition 344 (Costa Rica Seismogenesis Project), to constrain deformation mechanism operating in the subducting Cocos Plate. Cathodoluminescence studies, mechanical e‐twin piezometry and electron backscatter diffraction (EBSD) analyses of carbonate veins were used to give insights into the deformation conditions and to help to understand the tectonic deformation history of the Cocos Plate offshore Costa Rica. Analyses of microstructures in the sedimentary rocks and in the basalt of the igneous basement reveal brittle deformation, as well as crystal‐plastic deformation of the host rock and the vein material. Cathodoluminescence images showed that in the basalt fluid flow and related precipitation occurred over several episodes. The differential stresses, obtained from two different piezometers using the same parameter (twin density), indicate various mean differential stresses of 49 ± 11 and 69 ± 30 MPa and EBSD mapping of calcite veins reveals low‐angle subgrain boundaries. Deformation temperatures are restricted to the range from 170°C to 220°C, due to the characteristics of the existing twins and the lack of high‐temperature intracrystalline deformation mechanisms (>220°C). The obtained results suggest that deformation occurred over a period associated with changes of ambient temperatures, occurrence of fluids and hydrofracturing, induced differential stresses due to the bending of the plate at the trench, and related seismic activity.

## Key Points

- Mesoscale structures and microstructures of veins and host rocks imply increased differential stress conditions within the Cocos Plate
- Variation of microstructures within Hole U1414A indicates local changes in deformation regime and several fluid flows
- Deformation occurred over a period with changes of ambient temperatures and differential stress, occurrence of fluids and hydrofracturing

## 1 Introduction

Integrated Ocean Drilling Program (IODP) Expedition 344 as part of the Costa Rica Seismogenesis Project (CRISP) focused on sampling the lithology of the incoming and overriding plates, characterizing recent and paleo fluids, and measuring the ambient temperatures and stresses that lead to a transition from stable to unstable slip along the Cocos Plate‐Caribbean Plate boundary [*Harris et al*., 2013a]. Site 344‐U1414 (proposed Site CRIS‐19A), located on the subducting Cocos Plate, was drilled to investigate the lithostratigraphy and pore water of the sedimentary sequence and the uppermost portions of the underlying oceanic basement. Previous investigations showed that the Cocos Plate offshore Costa Rica has a complex tectonic history [*Meschede et al*., 1998; *Barckhausen et al*., 2001; *Brandstätter et al*., 2016]. The purpose of this paper is to constrain deformation mechanisms and required conditions in the upper crust of the Cocos Plate offshore Osa Peninsula and to get a better understanding of the deformation history at Site 344‐U1414 by the investigation of mineralized veins.

Veins can be helpful to unravel the deformation history of their host rocks and the effect of associated fluids [e.g., *Ramsay and Huber*, 1983; *Hilgers and Urai*, 2002]. The formation and composition of veins and deformation mechanisms operative in the vein material are important to obtain information about stress conditions, fluid sources and circulations, and chemical alteration processes [*Janssen et al*., 1998; *Gratier et al*., 2003; *Herwegh* *et al*., 2005]. Natural low strain deformation in calcite is documented by *Takeshita et al*. [2014] and *McNamara et al*. [2016] and experimental low strain deformation by *Rybacki et al*. [2013], characterized by brittle deformation (fractures) and mechanical e‐twinning. In comparison, examples of highly strained calcite microstructures include the work of *Bestmann and Prior* [2003], *Barnhoorn et al*. [2004], and *Herwegh et al*. [2005]. Dominant deformation mechanisms are, e.g., diffusion or dislocation processes and grain boundary migration recrystallization. To get information about the deformation mechanisms, we analyzed microstructures within lithified sedimentary rocks and the basalts at Site 344‐U1414, with focus on veins, especially calcite veins, by combining two mapping techniques, cathodoluminescence (CL) and electron backscatter diffraction (EBSD), with stress piezometry of mechanically formed e‐twins of calcite.

## 2 Geological Background

**Figure 1:**
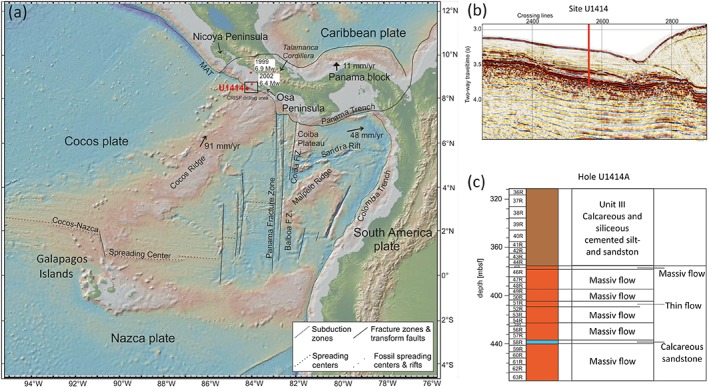
(a) Digital elevation map of the Costa Rica area (http://www.geomapapp.org) [*Ryan et al*., 2009], showing the location of drilling Site U1414 of IODP Expedition 344, location of seismic events (red stars) and the context to the general plate tectonic setting. F.Z.‐fracture zone and MAT‐Middle America Trench. (b) Seismic travel time section of Line 2497 with location of Site 344‐U1414 [after *Harris et al*., 2013b]. (c) Stratigraphy of lowest units of Site 344‐U1414. The lithified sedimentary rocks of Unit III and eight igneous basement units with one intercalated sedimentary layer compose 162 m of the cored drill hole [after *Harris et al*., 2013b].

The CRISP drilling area is characterized by the low‐angle subduction of the oceanic Cocos Plate beneath the Caribbean Plate offshore Costa Rica (Figure 1a). In this area the incoming plate shows large variations in bathymetry along strike and a fast convergence rate with 90 mm yr^−1^ [*DeMets*, 2001]. This active continental margin is a sediment‐poor subduction zone with a history of *M*_*w*_ > 7 earthquakes and active tectonic erosion [e.g., *Ranero and von Huene*, 2000; *Harris et al*., 2012; *Vannucchi et al*., 2013]. The recently documented earthquakes in this seismogenic zone are the 1999 Quepos (6.9 *M*_*w*_) and the 2002 Osa earthquakes (6.4 *M*_*w*_) offshore the Osa Peninsula (Figure 1a). The 2002 earthquake nucleated 40 km west of the Osa Peninsula and was a shallow underthrusting event [*Harris et al*., 2013a]. A significant influence on the tectonics of this area has the Cocos Ridge, a 2.5 km high topographic elevation on the Cocos Plate, which was formed by the passage of the Cocos Plate over the Galapagos hot spot. IODP Site 344‐U1414 is situated at the western margin of the Cocos Ridge, with 1 km distance to the Middle America Trench offshore the Osa Peninsula (Figure 1b) and is one of the two sites situated on the incoming plate. The second IODP Site 334‐U1381 is ~4.5 km seaward offshore the Osa Peninsula and Caño Island.

## 3 Lithostratigraphy and Sample Description

### 3.1 Sedimentary Cover

**Figure 2:**
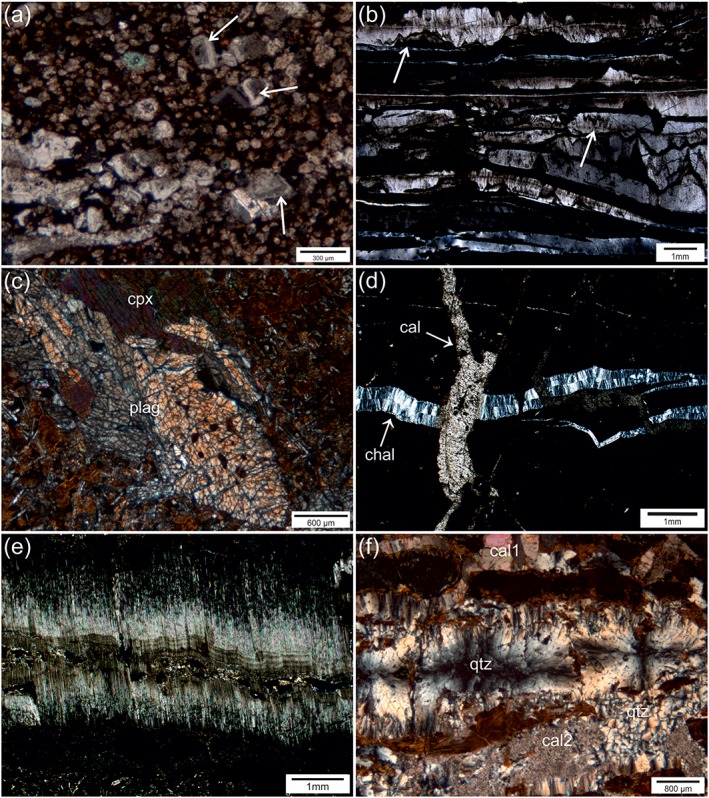
Microphotographs of the host rocks and veins of Unit III and the igneous Cocos Plate basement. (a) Zoned dolomite rhombs (arrows), calcite, and siliceous components forming the sedimentary host rock (sample JB36). (b) Bedding parallel stylolites (arrows) in calcite veins (sample JB40). (c) Microcracks transecting phenocrysts, plagioclase (plag), and clinopyroxene (cpx), in the altered basalt (sample JB73). (d) Veins in Unit III showing crosscutting relationships, vein filled by chalcedony (chal) are crosscut by younger calcite veins (cal) (sample JB43). (e) Antitaxial vein with fibrous and acicular calcite crystals and a median line consisting of wall rock fragments, clay minerals, and quartz (sample JB84). (f) Polymineralic vein composed of spherulitic quartz grains (qtz), surrounded by a clay selvage and blocky, occasionally elongated, older calcite (cal1) and younger calcite grains (cal2) replacing quartz in the lower part (sample JB74).

Hole U1414A comprises 375.5 m of sediment and 96.35 m of oceanic basement recovery [*Harris et al*., 2013a]. The sediments can be divided into three sedimentary units (Units I–III), whereby Units I and II are composed of unconsolidated sediments. At the boundary between Units II and III, a significant increase of lithification is noticeable [*Harris et al*., 2013a]. Unit III consists of strongly lithified calcareous and siliceous silt to sandstone (Figure 1c). Sedimentary structures, such as bedding and bioturbation, are preserved and nine tephra horizons occur as discrete layers or ash pod layers [*Harris et al*., 2013b]. Well‐preserved fossils like radiolarians occur in the sedimentary host rock. Components are mostly well rounded, vary in size, and consist of calcite, dolomite, and quartz. Some dolomite grains show a characteristic rhombohedral shape, with inclusion rich cores surrounded by a clear rim composed of calcite or silica, suggesting dedolomitization (Figure 1a, arrows indicate the described fabrics). Bedding and foliation planes are partially filled by silica and calcite precipitations, and the matrix consists mainly of calcite cement. Foliation planes can be distinguished from bedding planes due to observations of a flow of matrix around clasts and are the result of diagenesis and confining pressure. The foliation spacing and the thickness of the bedding layers vary from a few millimeters to centimeters within Unit III. Especially in core 344‐U1414A‐40R and in the upper part of core 344‐U1414A‐41R samples show a strong foliation and carbonate‐cemented stylolites with bedding and vein parallel orientation (Figure 2b, arrows). Faults, some of them being high‐angle normal faults [*Harris et al*., 2013a], occur at various dip angles throughout Unit III. Discrete and blocky calcite veins, partly associated with the fault planes, crosscut the lithified sediments. Hydrofracturing resulted in wall rock fragments embedded within the veins [*Brandstätter et al*., 2016]. Lithification of sediments of Unit III is mainly related to basaltic eruptions and advective heat transfer, as described in detail by *Brandstätter et al*. [2016]. Heat transfer additionally induced the mobilization of pore fluids, basically showing seawater chemistry, triggering first vein formation within Unit III [*Brandstätter et al*., 2016].

### 3.2 Oceanic Basement

The Cocos Ridge magmatic basement broadly comprises aphyric to highly phyric massive and thin basaltic flows, subdivided into seven units, with one additional unit of intercalated calcareous sandstone (Figure 1c). Igneous lithologic units were defined by changes in lava morphology, flow boundaries, texture, and phenocryst occurrence. Slight to high alteration is characterized by brecciation, partial replacement of groundmass mineralogy, partial to complete replacement of phenocrysts, especially olivine and clinopyroxene [*Harris et al*., 2013b]. Veins and vesicles are partially completely filled with clay minerals, quartz, carbonate, and pyrite. Microcracks in phenocrysts are common in the drill sections where veins are filled by quartz and the basalt shows an increased stage of alteration (Figure 2c).

## 4 Methods: Analytical Techniques

### 4.1 Cathodoluminescence (CL)

CL studies were carried out with a hot‐cathode‐CL microscope HC5‐LM, at the Institute of Earth Sciences (NAWI Graz Geocenter, University of Graz, Austria), in order to identify discontinuities in the luminescence intensity as CL banding and zonation. Excitation of luminescence on the surface of polished thin sections is enabled by electron bombardment with a voltage of maximum 14 kV and a filament current of <1.6 to 2.7 mA. Thin sections were coated with carbon serving as conductive layer. CL images are taken with 5X magnification and with an exposure time of 2.25 s from all calcite veins including blocky and discrete veins in the sedimentary layers of Unit III and from antitaxial, syntaxial, and composite veins in the basalt.

### 4.2 Piezometry and Deformation Temperature From Calcite Twinning

Twins in calcite grains are the result of crystal‐plastic deformation and can be used for paleostrain and related stress estimations [e.g., *Rowe and Rutter*, 1990; *Burkhard*, 1993; *Ferrill et al*., 2004]. The mechanical e‐twinning in calcite occurs at temperatures below 400°C [*Groshong*, 1988], depending on the orientation of stress and on the ability to overcome the critical resolved shear stress (CRSS) along one of the three *e* planes [*Jamison and Spang*, 1976; *Wenk et al*., 1987; *Burkhard*, 1993; *Lacombe and Laurent*, 1996; *Ferrill*, 1998]. The CRSS for calcite *e*‐twinning varies between 2 and 12 MPa, depending on grain size, mean stress, and temperature [*Turner et al*., 1954; *Jamison and Spang*, 1976; *Rowe and Rutter*, 1990; *Burkhard*, 1993; *Ferrill*, 1998]. We used an optical microscope (Leitz Laborlux 12 Pol) with an attached digital camera (Zeiss AxioCam ICc1) to measure twin width and twin density (twins per millimeter) in 14 samples. The thin sections were oriented parallel to the drill core axis. Mean twin width is determined by the average twin width for each twin set of a calcite grain. The twin density was obtained by counting the number of twins in one grain, normalized to a unit length of 1 mm for both piezometers. We were using the mean twin density value of each grain for both piezometry calculations.

Differential stress (Δ*σ*) was calculated after the equation of *Rowe and Rutter* [1990]:

(1) Δσ=−52.0+171.1logD

with Δ*σ* in megapascal and *D* is twin density (number of twins per millimeter).

The related experiments by *Rowe and Rutter* [1990] were performed in the temperature range from 200 to 800°C, and the standard error on the best fit line was 43 MPa.

A second method to determine the differential stress is the experimentally calibrated twin density piezometer after *Rybacki et al*. [2011] for low temperatures from 20 to 350°C:

(2) $$\Delta \sigma =10^{1.29\pm 0.02}{\rho_{\text{twin}}}^{0.50\pm 0.05}$$

Results are given in megapascal, and *ρ*_twin_ denotes the twin density (number of twins per millimeter). For details of the calibration of this piezometer, see Appendix A1 in *Rybacki et al*. [2011].

The process of mechanical twinning depends highly on the differential stress and grain size. The thickness of twins is mainly a function of deformation temperature and depends only to a minor degree on strain, strain rate, or stress [e.g., *Rowe and Rutter*, 1990; *Burkhard*, 1993; *Ferrill et al*., 2004]. The classification of the twin morphology can be used as a geothermometer for low‐grade metamorphic conditions and four types of calcite twins can be differenced: Type I twins are thin (<1 μm), straight, and evolve at <170 to 200°C; type II twins are thicker (≫1 μm) and slightly lensoid, formed at 150 to 300°C; type III twins are curved, tapered thick twins, formed at temperatures >200°C; and the last type IV twins are thick, patchy, irregular, accompanied by grain boundary migration and occur at temperatures >250°C [*Burkhard*, 1993; *Ferrill et al*., 2004].

### 4.3 Electron Backscatter Diffraction (EBSD) Analysis of Vein Calcite

EBSD mapping was used to analyze microstructures of two different types of calcite veins in the sedimentary layers of Unit III. This method should help to reveal the deformation history of the veins. EBSD measurements were accomplished at the University of Vienna, Department for Lithospheric Research, with a Quanta 3‐D FEG device equipped with an EDAX Pegasus Apex 4 system, consisting of a Digiview IV EBSD camera and an Apollo XV silicon drift detector for energy‐dispersive X‐ray spectrometry. Samples were chemomechanically polished with Köstrosol 3530. Two samples were analyzed, JB39 and JB58, with a working distance of approximately 14 mm, samples tilted at 70°, an acceleration voltage of 15 kV, and a beam current of 4 nA. The step size was 1.9 μm and 5.5 μm, respectively. EBSD data were processed using the OIM analysis software; misorientations axes were plotted in contoured inverse pole figures (IPF) using the MATLAB© toolbox MTEX [*Bachmann et al*., 2010].

## 5 Results

### 5.1 Veins

Veins are heterogeneously distributed within the lithified sedimentary rocks of Unit III and the oceanic basement. The vein orientation is highly variable throughout both units. Monomineralic calcite veins and subordinate quartz veins occur in the sedimentary rocks of Unit III from approximately 345 to 375 m below sea floor (bsf). Different vein types and generations can be distinguished by their morphology and crosscutting relationships [*Brandstätter et al*., 2016]. Unit III comprises discrete veins of calcite and subordinate discrete veins with different growth intervals of quartz, irregular, blocky veins with coarse‐grained calcite and wall rock fragments embedded within the vein filling and some small branched, irregular veins filled with calcite. Locally, calcite veins crosscut discrete quartz veins (Figure 2d). Microstructures in the vein calcite comprise few bedding and vein parallel stylolites, abundance of twins, undulose extinction, and small number of subgrains.

In the 96.35 m of the recovered oceanic basement, a total number of 1159 veins were recorded. Average vein density is 19.99 veins per meter of recovered core; vein fill makes up 3.5% by volume of the recovered core [*Harris et al*., 2013a]. Vein thickness ranges from <0.1 mm to 1.5 cm. Vein morphology is highly variable with morphologies that range from planar, curved, branching, anastomosing, irregular, en echelon arrangement, and crosscutting. Vein minerals comprise clay minerals, quartz, calcite, aragonite, and pyrite. Veins are mainly polymineralic and contain a combination of any of the aforementioned filling phases. Minor veins (5.8%) are flanked by halos. Veins of clay minerals are crosscut by all other mineral phases. Slickenlines within saponite in some of the larger veins suggest precipitation during vein opening accompanied with displacement [*Harris et al*., 2013b].

According to their growth morphology, the veins were classified following the descriptions by *Bons et al*. [2012] and divided into three different types: (a) antitaxial veins with fibrous and acicular calcite crystals and a median line formed by coarse, blocky quartz aggregates, and wall rock fragments (e.g., samples JB81, JB84, and JB88) (Figure 2e); (b) polymineralic‐filled veins surrounded by clay minerals along the vein wall, with several generations of calcite; the first calcite generation is partly displaced by spherulitic quartz, and the latest generation of calcite occurs as small idiomorphic grains overlying the quartz (e.g., samples JB60, JB74, and JB78) (Figure 2f); and (c) syntaxial veins, filled with elongated crystals of aragonite with a clay selvage (e.g., samples JB59, JB62, JB63, JB66, and JB69) [*Brandstätter et al*., 2016]. Vesicles occur discretely throughout the units in the basalt, and their abundance varies within the different units. The common vesicle‐filling phases are clay minerals, pyrite, quartz, and calcite. Microstructures in the carbonate veins (calcite and aragonite) are characterized by undulose extinction. Crack‐seal processes contributed to the formation of composite veins. Quartz is nearly undeformed except of the rarely occurrence of microcracks.

### 5.2 Cathodoluminescence

**Figure 3:**
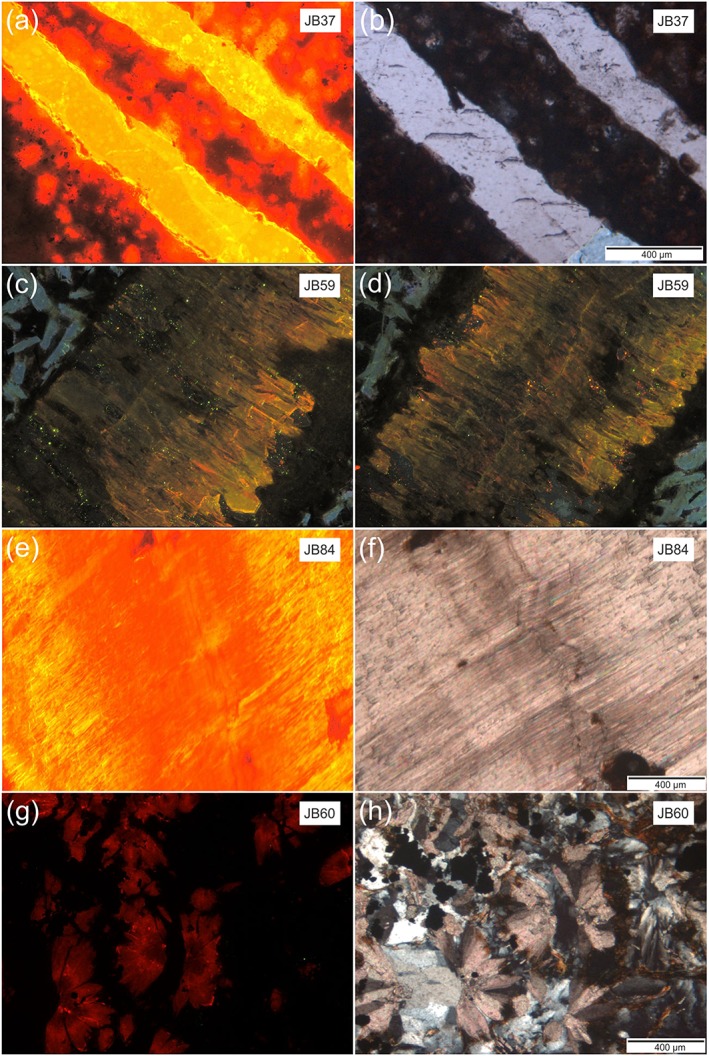
Cathodoluminescence images and microphotographs of various samples. (a and b) Homogenous calcite veins of sample JB37. (c) Syntaxial and (d) antitaxial growth of aragonite vein in the Cocos Ridge basalt (sample JB59). (e and f) Antitaxial calcite vein with characteristic median line in the center (sample JB84). (g and h) Spherulitic calcite grains fractured and displaced by quartz grains and pyrite (sample JB60).

CL studies can help to clarify the formation history of the different carbonate veins in the sedimentary rocks and in the basalt. The different luminescence properties of the carbonates depend on the abundance of manganese (Mn^2+^) and rare earth elements, which appear to be the main activator, and on iron (Fe^2+^), which serves as inhibitor [e.g., *Medlin*, 1968; *Marshall*, 1988; *Machel*, 2000; *Richter et al*., 2003]. The intensity of CL emission depends mainly on the incorporation of Mn^2+^ ions in the lattice of calcite, resulting in the luminescence colors orange yellow, yellow orange, and orange and yellow green; green are the luminescence colors of aragonite. Extinction occurs due to Fe^2+^ quenching and Mn^2+^ self‐quenching at high Mn^2+^ concentrations [e.g., *Marshall*, 1988; *Machel*, 2000; *Habermann et al*., 2000; *Richter et al*., 2003]. This implies that the differences in CL intensity are either bound to changes in geochemistry and temperature of the fluid the crystals precipitated from to variation in the crystal growth rate or are deformation related [*Rye and Bradbury*, 1988; *Urai et al*., 1991]. During deformation, primary CL pattern can be destroyed, e.g., due to new grains formed by grain boundary migration. The CL images of the sedimentary rocks of Unit III show that cements consist of a carbonate phase as well as a nonluminescent phase (Figure 3a). Calcite veins from Unit III display a lack in variation of CL patterns, despite from the small brighter spots, which were generated by fluid inclusions (dark spots in Figure 3b) The veins are surrounded by a material showing the same intense red luminescence color as some material in the host rock (Figure 3a).

Aragonite veins are totally absent in the sedimentary rocks; they are rare in the basalt and restricted in the uppermost part of the igneous basement. The aragonite vein of sample JB59 consists of mostly unitaxial syntaxial crystals (Figure 3c). Syntaxial veins can form from a single cracking event or from dissolution by addition of material on a single plane. This overgrowth on vein crystals can be both on sides or on one side (unitaxial) of the growth plane [*Bons et al*., 2012]. In distinct parts of the aragonite vein, however, the symmetrical CL banding in the center of the vein and the symmetrical extending of the fibers toward the vein wall are a good evidence for antitaxial growth (Figure 3d). Antitaxial growth is until now not fully understood; one assumption is that antitaxial veins formed by crack‐seal mechanisms [e.g., *Passchier and Trouw*, 2005; *Barker et al*., 2006]; an alternative conjecture is that antitaxial growth is enabled on essentially closed interfaces between vein and wall rock [e.g., *Bons and Montenari*, 2005]. *Hilgers and Urai* [2002] showed that antitaxial veins are formed by accretion of material at the vein‐wall interface, with the absence of growth competition and absence of nucleation of new grains.

Also, the fibrous calcite vein of sample JB84 (Figures 3e and 3f) is characterized by antitaxial crystal growth, showing a CL banding aligned parallel to the median line. The parallelism of the median line and CL banding of the antitaxial veins indicates the same growth rate of the fibrous calcite crystals [*Bons and Montenari*, 2005].

The polymineralic veins in the basalt are characterized by several crack‐seal events and different growth phases of carbonates, displayed by discontinuities in the CL intensity pattern. Fibrous calcite and radiating columnar calcite have filled multiple fractures, and it is difficult to determine the sequences of fracturing. The spherulitic calcite grains of sample JB60 are fractured and displaced by spherulitic nonfractured quartz grains and pyrite (Figures 3g and 3h and Table 1).

**Table 1:** Sample Codes; the Related IODP Sample Requests Are 1607IODP and 1911IODP

Sample #	Sample Code IODP Expedition 344	Depth	Lithology	Veins	Methods
JB33	344‐U1414A‐40R‐1‐W 6/9‐	345.58	Sedimentary cover	calcite	twins
JB37	344‐U1414A‐41R‐1‐W 8/10‐	355.30	Sedimentary cover	calcite	CL, twins
JB39	344‐U1414A‐41R‐1‐W 23/28‐	355.45	Sedimentary cover	calcite	twins, EBSD
JB40	344‐U1414A‐41R‐2‐W 12/13‐	356	Sedimentary cover	calcite	twins
JB41	344‐U1414A‐41R‐2‐W 57/63‐	356.50	Sedimentary cover	calcite	twins
JB42	344‐U1414A‐41R‐2‐W 69/72‐	356.65	Sedimentary cover	calcite	twins
JB43	344‐U1414A‐41R‐2‐W 73/77‐	356.70	Sedimentary cover	calcite	twins
JB46	344‐U1414A‐41R‐2‐W 93/99‐	356.90	Sedimentary cover	calcite	twins
JB50	344‐U1414A‐42R‐1‐W 13/15	360.20	Sedimentary cover	calcite	
JB51	344‐U1414A‐42R‐1‐W 21/24‐	360.35	Sedimentary cover	calcite	twins
JB52	344‐U1414A‐42R‐1‐W 31/36‐	360.45	Sedimentary cover	calcite	twins
JB53	344‐U1414A‐42R‐1‐W 39/40	360.50	Sedimentary cover	calcite	twins
JB57	344‐U1414A‐44R‐1‐W 12/14‐	369.95	Sedimentary cover	calcite	twins
JB58	344‐U1414A‐44R‐1‐W 21/24‐	370.05	Sedimentary cover	calcite	twins, EBSD
JB59	344‐U1414A‐45R‐2‐W 3/5‐	375.98	Oceanic basement	aragonite	CL
JB60	344‐U1414A‐45R‐2‐W 56/64‐	376.53	Oceanic basement	quartz, calcite	CL
JB62	344‐U1414A‐46R‐2‐W 32/34‐	376.32	Oceanic basement	aragonite	
JB63	344‐U1414A‐46R‐3‐W 86/90‐	379.95	Oceanic basement	aragonite	
JB66	344‐U1414A‐48R‐1‐W 88/92‐	390	Oceanic basement	aragonite	
JB69	344‐U1414A‐49R‐1‐W 105/111‐	394.98	Oceanic basement	aragonite	
JB73	344‐U1414A‐51R‐3‐W 128/134‐	407.21	Oceanic basement	quartz, calcite	
JB74	344‐U1414A‐53R‐1‐W 126/131‐	414.5	Oceanic basement	quartz, calcite	
JB78	344‐U1414A‐57R‐1‐W 38/43‐	433.20	Oceanic basement	quartz, calcite	
JB81	344‐U1414A‐57R‐1‐W 71/76‐	433.55	Oceanic basement	calcite	
JB84	344‐U1414A‐57R‐2‐W 46/52‐	434.55	Oceanic basement	calcite	CL
JB88	344‐U1414A‐61R‐1‐W 45/49‐	452.56	Oceanic basement	calcite	

### 5.3 Calcite Twinning

Twins occur mainly in calcite veins within the sedimentary layers of Unit III. The fibrous or small recrystallized calcite grains within the basalt are not twinned, one aragonite vein (sample JB62), however, contains twins. Twin density and the mean twin width of calcite grains were measured at 14 samples of Unit III.

**Figure 4:**
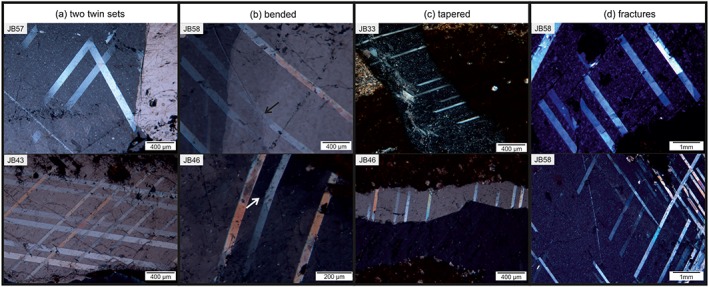
Microphotographs of different shapes and occurrences of calcite twins. (a) Samples JB57 and JB43 containing two twin sets of type II twins. Characteristics of deformations twins: (b) bended twins and subgrain boundaries (arrows, samples JB58 and JB46) and (c) tapered endings (samples JB33 and JB46). (d) Calcite twins commonly end at microcracks (sample JB58).

Generally, twins in calcite result from mechanical *e*‐twinning, due to the low CRSS along the twinning plane, but they can also develop during stress release after deformation or under cooling effects [*Lacombe and Laurent*, 1996]. Deformation twins in the samples show one or more twin sets; the interface is commonly curved or tapered and the shape sometimes bended. The most abundant twin type, after the classification of *Burkhard* [1993], is type II; type I twins may appear in few samples. Two twin sets are common (Figure 4a), occasionally with two different twin types. Type II twins show a thickness from 2.9 to 106 μm, with straight or weakly bended twin boundaries (Figure 4b). Some of these twins show tapered endings (Figure 4c) or often end at microcracks (Figure 4d). According to *Burkhard* [1993], type II twins are indicative for deformation temperatures between 150 and 300°C.

**Figure 5:**
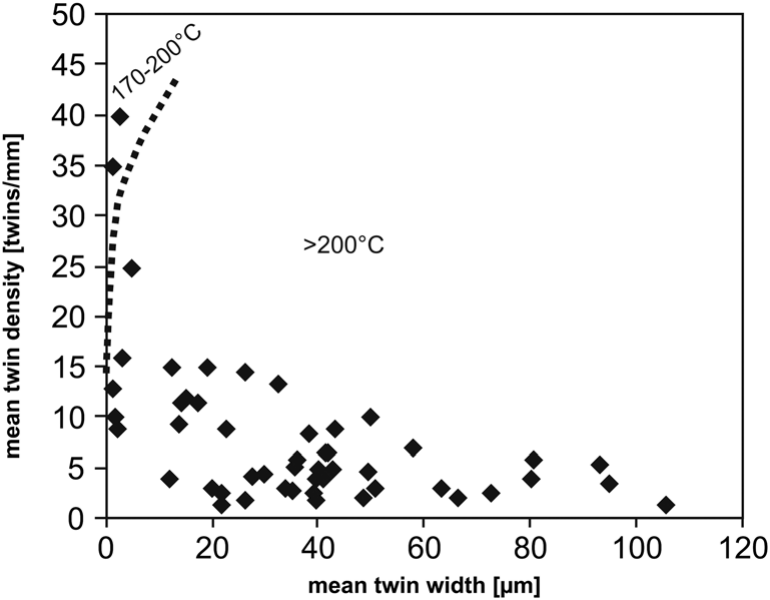
Graphs of twin parameters: the total number of twin data is plotted in the graph after *Ferrill et al*. [2004] with two fields of deformation temperature.

**Figure 6:**
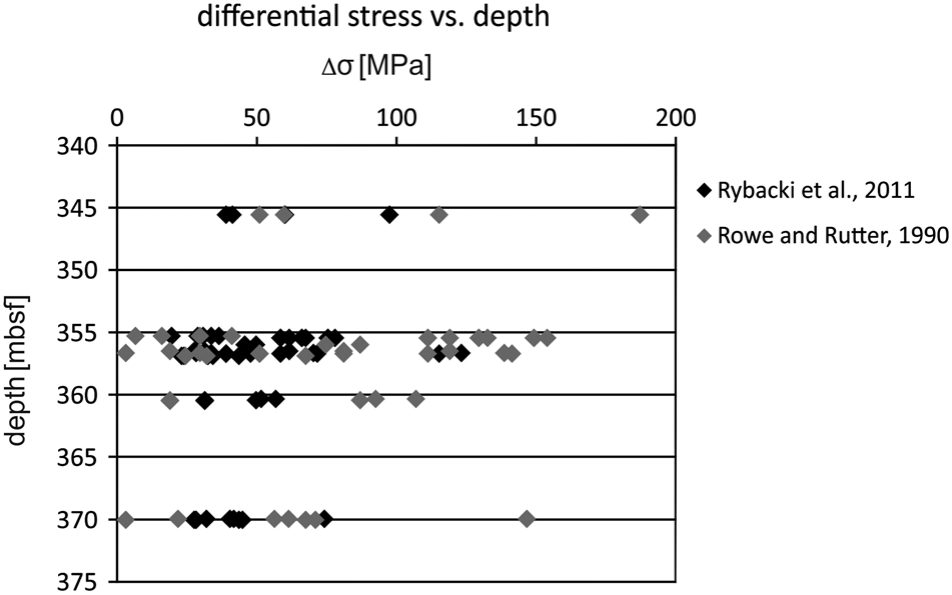
Differential stress data after *Rybacki et al*. [2011] (black rhombs) and after *Rowe and Rutter* [1990] (grey rhombs).

Crossplot diagrams of twin density with twin width can provide additional information about the temperature of deformation, since twin width and deformation temperature are positively correlated [*Ferrill et al*., 2004]. *Ferrill et al*. [2004] found out that strain aggregation at temperatures above 200°C is accomplished by formation of thick twins and enlargement of existing twins, whereby at temperatures below 170°C the formation of new thin twins rather than formation of thick twins or twin enlargement is favored. The samples of this study show mainly low twin densities and increased twin widths, indicating temperatures of deformation exceeding 200°C, two values plot in the field 170–200°C (Figure 5). Twin densities of vein calcite deformed above 200°C do not exceed 30 per millimeter but can reach a mean twin width of about 106 μm. The differential stress varies between 0.2 and 187 MPa (Figure 6), with a mean value of 69 ± 30 MPa, according to *Rowe and Rutter* [1990]. Values indicating a deformation temperature below <200°C are excluded for differential stress estimations [*Rowe and Rutter*, 1990], because this technique is not suitable for low‐temperature deformation of calcite [*Ferrill*, 1998]. The calculations of the twin density piezometer of *Rybacki et al*. [2011] obtained a mean differential stress of 49 ± 11 MPa with a range from 24 to 123 MPa (Figure 6 and Table 2).

**Table 2:** Results of Calcite Twins Analyses

Sample	Depth (m bsf)		Twin Width (μm)	Twin Density (mm^−1^)	n Grains	Δ*σ* Rybacki (MPa)	Δ*σ* R&R (MPa)
JB33	345.58	range	5–30	4–25	4	39–97	51–187
		average	30 ± 5	11 ± 5		59 ± 14	103 ± 31
JB39	355.45	range	2–44	9–16	9	58–78	111–154
		average	14 ± 4	12 ± 1		67 ± 3	132 ± 5
JB40	356	range	42–94	5.5–6.5	2	46–50	75–87
		average	68 ± 18	6 ± 1		48 ± 2	81 ± 6
JB41	356.5	range	27–64	2–10	4	28–62	0–119
		average	44 ± 7	5 ± 2		43 ± 8	58 ± 26
JB42	356.65	range	1–40	2–40	4	28–123	30–139
		average	20 ± 8	15 ± 9		64 ± 22	56 ± 42
JB43	356.7	range	2–81	4–13.5	7	39–115	51–141
		average	43 ± 10	11 ± 4		59 ± 11	81 ± 16
JB46	356.9	range	22–106	1.5–5	5	24–44	0–68
		average	48 ± 13	3 ± 1		33 ± 3	32 ± 14
JB51	360.35	range	39–58	7–8.5	2	52–57	93–107
		average	48 ± 7	8 ± 1		54 ± 3	100 ± 7
JB52	360.45	range	39–42	2.5–6.5	2	31–50	16–87
		average	34 ± 1	5 ± 2		40 ± 9	52 ± 36
JB53	360.5	range	22	3	1	34	30
		average	22 ± 0	3		34	30
JB57	369.95	range	27—73	2.5–14.5	4	31–74	16–147
		average	44 ± 9	6 ± 3		46 ± 10	68 ± 28
JB58	370.05	range	36–67	2–5	4	28–44	0–68
		average	49 ± 6	4 ± 1		36 ± 5	34 ± 19

### 5.4 Electron Backscatter Diffraction

The presence of deformation structures, such as undulose extinction and subgrain boundaries, indicates intracrystalline‐plastic deformation by dislocation creep [e.g., *Twiss*, 1977; *Wheeler et al*., 2001]. Such features can be predominantly observed in samples of Unit III; subgrains are totally missing in the calcite veins in the basalt. Detailed analysis of misorientation patterns has been performed to constrain the microfabric forming processes.

The samples selected for the EBSD analysis show different deformation patterns, although the vertical distance between the veins does not exceed 15 m (Table 2). A calcite vein of sample JB39 contains twins and undulose extinction with minor subgrain boundaries. The fine‐grained host rock shows a moderate to strong foliation. The host rock of sample JB58, a coarse‐ to fine‐grained calcareous limestone with silicic cement, consists of calcite veins with deformation twins and subgrains overlain by bended twins (Figure 4b, arrows).

**Figure 7:**
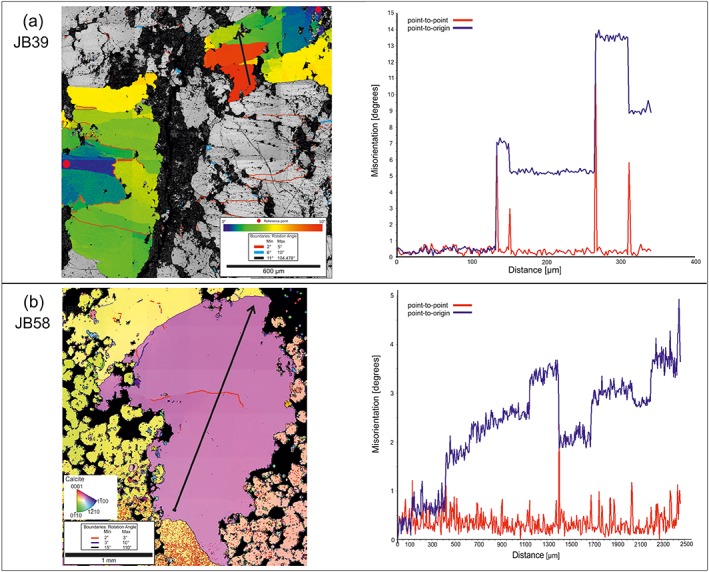
Results of EBSD mapping. (a) Vein calcite of sample JB39 contain several grains, two are displayed with a color gradient with a tolerance angle of 10°, and red point represents the reference point. The black arrow indicates the direction of the misorientation profiles. Misorientation profile trough the marked grain, blue line represents point to origin and the red line point to point misorientation angles. (b) EBSD data of sample JB58. Color coded map (inverse pole figure map typ) of the vein with low‐angle boundaries (red and blue lines) and high angle boundaries (black line). The calcite grain in the center is transected by a profile (black arrow) and (right) the corresponding misorientation profile, showing low‐angle boundaries.

The blocky calcite grains of sample JB39 show a misorientation of up to 14° (Figure 7a). The misorientation profile and the EBSD map, presenting the misorientation gradient by color coding (Figure 7a), indicate a distinct change in crystal‐lattice orientation at subgrain boundaries, while the lattice orientation across each subgrain remains almost constant.

**Figure 8:**
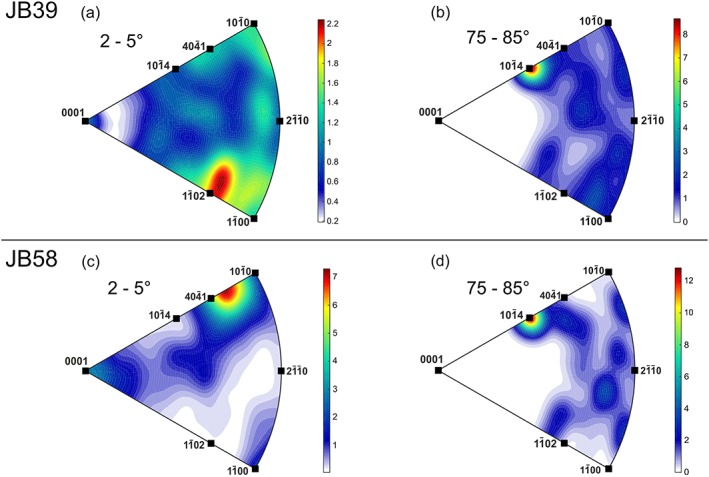
(a) Misorientation axes displayed in countered IPF for misorientations of 2° to 5° showing a dominant maximum around {*f*} and (b) for misorientations from 75° to 85° showing maximum around {*r*} (sample JB39). (c) Misorientation axes displayed in countered IPF for misorientations of 2° to 5° showing a dominant maximum around <404®1> and (d) for misorientations of 75° to 85° showing a maximum around {*r*} (sample JB58).

The calcite grains in the vein of sample JB58 are coarser grained (average > 4 mm, Figure 7b), and the subgrains in this sample (average grain size of 548 μm) seem less evolved when compared to sample JB39, with misorientation angles of up to 5°. The misorientation profile shows a constant increase in misorientation angle with greater distance from the starting point. Major jumps in misorientation angle such as at a distance of ~1400 μm are associated with low‐angle grain boundaries. In order to put constraints on possible active slip systems, misorientation axes for misorientation angles from 2° to 5° and 75° to 85° were plotted in the calcite reference frame (hexagonal system) as IPF (Figures 8a–8d). For low angles, sample JB39 shows a weak maximum in orientation distribution function density (ODF maximum) of 2.2, normal to one of the *f* planes and a minor increase in ODF maximum (up to 1.8) around {*m*} (Figure 8a). Low‐angle grain boundaries with a misorientation angle of up to 10° result in elongated subgrains with an average size of 570 μm.

Misorientation axes for low angles in sample JB58 show a pronounced maximum (ODF maximum = 7.3) parallel to <404®1> and a weaker maximum around {*c*} (ODF maximum ~3.8, Figure 8c).

## 6 Discussion

Vein microstructures in the Cocos Ridge basalt and in the overlying sedimentary rocks reveal the deformation history of the Cocos Ridge at Site 344‐U1414 from its formation, movement toward and arrival at the Middle America Trench.

### 6.1 Deformation Structures and Fluid Flow

Mesoscale structures in the sedimentary rocks of Unit III (foliation, stylolites, and veins) indicate that the effective stresses were sufficient to trigger fluid‐assisted pressure solution followed by the formation of fractures and veins, subsequent to the lithification of the sedimentary rocks within Unit III. Paleopressures in the range of 20 MPa, related to the hydrostatic and lithostatic load, would be insufficient to cause substantial lithification as observed in Unit III and therefore require lithification processes that were facilitated by heat advection (see *Brandstätter et al*. [2016] for details). Fluid pressure enabled subsequent hydrofracturing, as indicated by blocky veins containing embedded host rock fragments. Effective fluid pressures can only form in a low permeability system, which prevents rapid upward migration of the overpressured fluid [*Sibson et al*., 1975]. The unconsolidated sediments (pelagic clay and nannofossil ooze) overlying the lithified sedimentary rocks of Unit III, potentially acted as a barrier that provided a low permeability regime. The CL images exhibit homogenous filling without any zoning of crystals of the veins in Unit III, suggesting one fast precipitation event [*Gratier and Gamond*, 1990], but it could also be the result of precipitation under isochemical conditions due to the buffering by the carbonate host rock [e.g., *Gao et al*., 1992; *Suchy et al*., 2000] and a homogenization of the fluids before precipitation. The second interpretation is more likely due to the occurrence of different growth generations of calcite in the basalt. The vein calcite in the basalt shows various manganese contents, due to the lack of calcite in the matrix and therefore less buffering. Quartz and aragonite are additional filling phases and different alteration states of the igneous host rock implying fluid flow occurrence over several episodes. The CL results combined with microscopic observations indicate that both host rocks most likely have seen multiple veining events.

Mineral phases of the hydrothermal veins in Unit III and in the basalt expose different microstructures such as fractures, twins, undulose extinction, and incipient subgrain formation. These structures indicate that the veins underwent (semi) brittle to crystal‐plastic deformation mechanisms during the movement of the Cocos Plate with different deformation conditions near the spreading center and possible hot spots and near the trench axis. Fractures in the basalt and microcracks in phenocrysts are probably related to local stress changes, induced by differential thermal contraction of phases in the cooling basalt [*Richter and Simmons*, 1974], and also due to subsequent reheating events [*Brandstätter et al*., 2016] and constitute the oldest deformation features. However, temperatures remained within a range that was not adequate for plastic deformation mechanisms within the basalt phenocrysts.

### 6.2 Piezometry and Temperature of Deformation

Mechanical *e*‐twinning occurred mainly in the blocky calcite veins in Unit III, rather than in the fibrous calcite veins of the basalt. Calcite twin piezometry yielded mean differential stresses of 49 ± 11 and 69 ± 30 MPa. During deformation of calcite, twinning occurred prior to dislocation climb due to a low CRSS, allowing for activation of mechanical twinning at low temperatures [*De Bresser and Spiers*, 1997]. Compared with the study of *Chen et al*. [2011], these values lie more or less in the field of stresses obtained from recrystallized greenschist facies marble (mean values 60–70 MPa). The obtained differential stresses are in the range of experimentally determined compressive strengths of carbonate rocks (several tens to approximately 125 MPa) as well as basic and ultrabasic rocks (approximately 70–170 MPa) and exceed common tensile strength values of carbonates and magmatic rocks (approximately 5–10 MPa) [e.g., *Cai*, 2010; *Yavuz et al*., 2013]. Implying additional fluid pressures, the resulting effective stresses were therefore sufficient to cause failure of the upper part of the Cocos Plate during plate convergence.

*Rowe and Rutter* [1990] and *Rybacki et al*. [2011] pointed out that the relationship between twin density and stress are independent of grain size; the obtained data of this study confirm this assertion. Although both equations are dealing with the identically measured twin density, the laboratory experiments were performed in two different regimes: (1) semibrittle regime: 20–350°C, 300–400 MPa, and strain rates of ~10^−4^ s^−1^ [*Rybacki et al*., 2011]; and (2) ductile regime: 200–800°C, 100–200 MPa, and strain rates from 10^−3^ to 10^−7^ s^−1^ [*Rowe and Rutter*, 1990]. Twinning is the result of deformation, and the number of twins is basically a function of strain. At higher temperatures other slip system can be activated and interfere with *e*‐twinning [*Burkhard*, 1993]; hence, the regime is significant. The differential stress calculations following the equation after *Rowe and Rutter* [1990] vary massive with small changes in twin density. Hence, the twin density sensitivity and the differential stress overestimation for calcite deformed at low temperature (<200°C) [*Ferrill*, 1998] make the piezometry calculation less viable, especially for high twin densities and result in differential stresses with large standard errors. Our results confirm the assumption of *Rybacki et al*. [2011] that *Rowe and Rutter*'s [1990] piezometer may be not suitable for application to naturally deformed rocks and confirm also *Burkhard*'s [1993] concerns to extrapolate the laboratory conditions to nature, due to the high main standard error and that an increasing number of twins result in excessive high differential stresses. *Rybacki et al*. [2011] compared the obtained stress estimations with a dislocation density piezometer, with satisfying results. The application of *Rybacki et al*. [2011] piezometer, calibrated at low temperatures, is therefore more appropriate for the low‐grade deformed calcite veins of this study.

Fluid inclusion microthermometric data document an anticlockwise pressure‐temperature evolution during vein precipitation and modification by isobaric heating and subsequent cooling at pressures between 21 and 35 MPa [*Brandstätter et al*., 2016]. Heat advection, related either to the Cocos‐Nazca spreading center or to hot spot activity closer to the Middle America Trench, led to isobaric heating, fluid overpressures, hydraulic fracturing, and vein formation [*Brandstätter et al*., 2016] previous to the onset of twin and subgrain formation. Although previous fluid inclusion studies [*Brandstätter et al*., 2016] would suggest an upper temperature limit of approximately 400°C for the evolution of the deformational microstructures, and the microstructural observations constrain the deformation temperatures lower than 220°C. Temperatures higher than 220°C allow dynamic recovery in calcite and would result in subgrain rotation, grain boundary migration, and formation of type III and IV twins [*Burkhard*, 1993; *Herwegh et al*., 2005; *Ebert et al*., 2007]. *Kennedy and Logan* [1998] demonstrated that the combination of small grain sizes and fluid‐grain interactions can lead to dislocation glide and grain boundary migration recrystallization at deformation temperatures lower 180°C. Although the presence of fluids is indisputable, the majority of our samples are composed of coarse‐grained calcite. We therefore presume that deformation occurred below 220°C due to the lack of dynamic recrystallization. The lower limitation of deformation temperature of 170°C is constituted by the twin parameters (Figure 5).

### 6.3 Intracrystalline‐Plastic Deformation Sequence of Calcite

**Figure 9:**
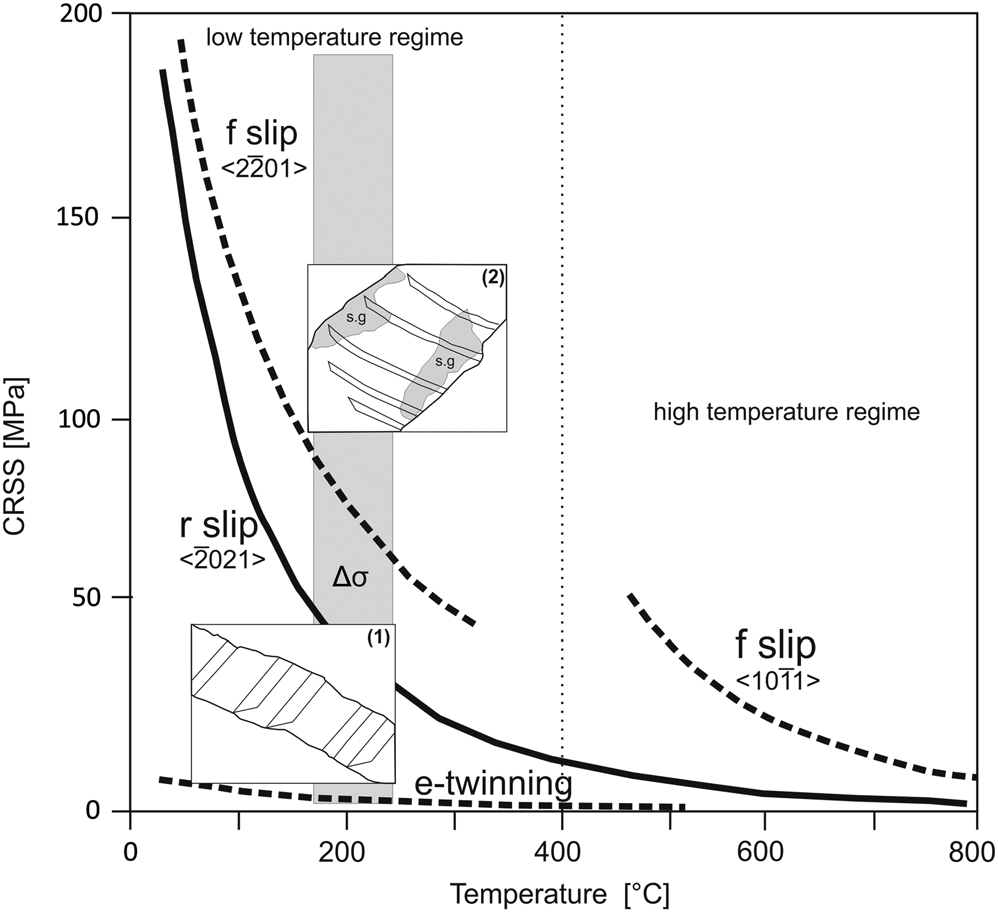
Diagram after *De Bresser and Spiers* [1997] showing a summary of the obtained intracrystalline deformation mechanism of calcite. The grey box displays the range of obtained differential stresses of the two piezometers; the corresponding range of deformation temperature was acquired from different twin parameters according the classifications after *Ferrill et al*. [2004] and *Burkhard* [1993]. Mechanical e‐twinning occurs at low CRSS and at proportional low temperatures (1). By exceeding the *f* and *r* slip line, subgrain formation is enabled (2).

The vein microstructures revealed from Site U1414 document a continuous evolution from the formation of mechanical twinning (Figure 9, 1), subgrains, indicating dislocation creep and further mechanical twinning (Figure 9, 2). The local overlap of twins and low‐angle boundaries and subgrains (dynamic) (Figure 9, 2) suggests that twinning continued throughout deformation, with overprinting subgrains.

The general evolution of deformation microstructures shows that subgrains and mechanical twins interact; both are finally transected by distinct microcracks. The beginning of subgrain formation indicates that deformation occurred at still comparatively higher temperatures allowing dislocations to climb. Actually, a greater strain rate can result in the activation of different slip systems, too, but to activate dislocation climb processes, the temperature should be appropriate. Proportionally high strain rates and differential stresses, however, can be expected adjacent to the Middle America Trench due to the rather high subduction rates in the range of 90 mm and the flat subduction angle offshore Costa Rica [*DeMets*, 2001]. The onset of subgrain formation therefore indicates the effect of either temperature reducing the CRSS or increased strain rate and/or differential stresses, resulting in the activation of additional calcite slip systems. According to *Trépied et al*. [1980], two end‐members can be described resulting in the development of characteristic misorientation axes. Screw dislocations with misorientation axes perpendicular to the slip plane and edge dislocations with misorientation axes that lie within the slip plane and perpendicular to the slip direction [*Lloyd and Freeman*, 1991; *Neumann*, 2000]. The observed misorientation axes normal to one of the *f* planes in sample JB39 can be explained by screw dislocations on {*f*}, being related to either a low‐temperature regime with slip in <202®1> direction or a high‐temperature regime with slip in <101®1> direction [*De Bresser and Spiers*, 1997]. The notable maximum about 75° to 85° in both samples (Figures 8b and 8d) is due to the *e*‐twinning system in calcite [*Bestmann and Prior*, 2003; *Valcke et al*., 2006]. Both samples show clearly a difference in dominant misorientation axes and therefore indicate the activation of different slip systems. The activation of these slip systems is temperature and strain rate dependent [*De Bresser and Spiers*, 1993, 1997].

Taking the upper temperature limit of approximately 220°C for the observed subgrains, and a lower temperature limit of approximately 170°C as the conditions during mechanical twinning, and assuming constant differential stresses as derived from piezometry during bulk deformation, the obtained subgrain misorientations suggest a low‐temperature slip regime on {*f*} for sample JB39, and probably also the easier low‐temperature *r* slip system was activated (Figure 9a). As it is known, the natural strain rates at the Middle America Trench in the study area are much less than the experimental strain rates provided by *De Bresser and Spiers* [1997] and therefore would require either higher temperatures or higher differential stresses for the activation of the high‐temperature slip system. Although the differential stresses are not well constrained for the discussed temperature interval, the activation of a high‐temperature f slip system can be excluded due to the aforementioned upper temperature limit of 220°C. The observed rotation axes around <404®1> for sample JB58 cannot be explained by either of the two end‐member models and described slip systems in calcite. The *r* slip system, the typical low‐temperature slip system in calcite [*De Bresser and Spiers*, 1993, 1997], could also be considered as a dominant slip system in these samples. The variation in subgrain size observed for the different samples can be related to local variations in differential stress [*Bestmann et al*., 2000; *Valcke et al*., 2007]. In distinct samples twins crosscut subgrains, in the area where twins and subgrains overlap, the twins show a bended shape (Figures 4b and 9a, 2). The variation of microstructures for the different samples together with the associated variation in dominant slip systems implies local changes in deformation conditions within small vertical distances. This can be attributed to the differing mechanical properties, and therefore deformational behavior, of the vein bearing host rock (Cocos Ridge basalt versus lithified sediments and different lithification, components, and cementation states within distinct sedimentary layers). This is basically associated with varying rheology contrasts between the host rocks and the veins, being presumably higher within the rigid basalts than within the mechanically much softer sediments and therefore with a local variation of stress concentrations and stress transfer across the vein‐wall rock interfaces.

### 6.4 Tectonic History

**Figure 10:**
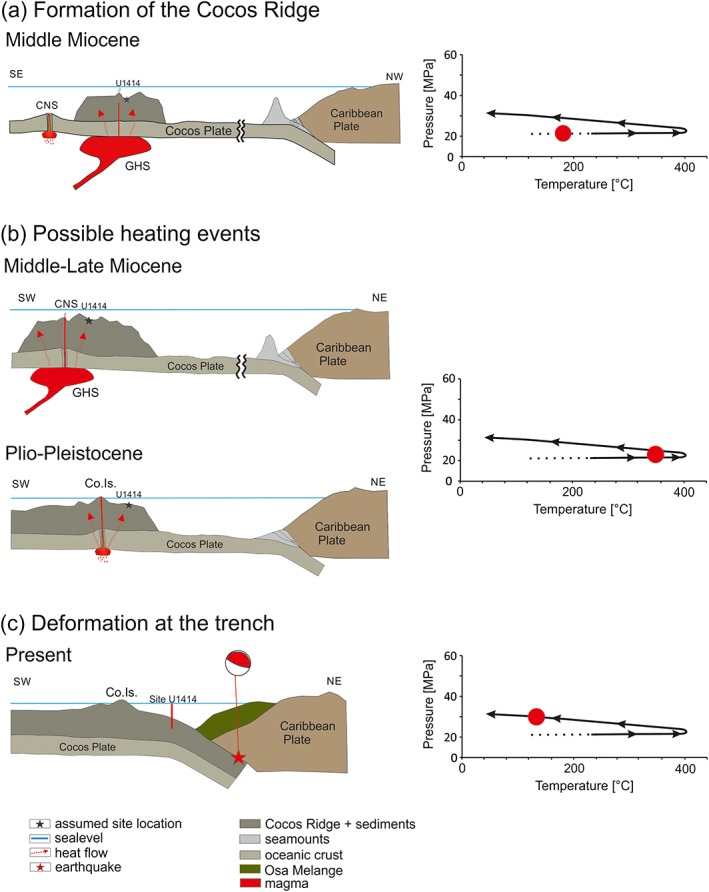
Diagram of the tectonic evolution of the study area compared with previous fluid inclusion results, modified after *Brandstätter et al*. [2016]. (a) In the middle Miocene the formation of the Cocos Ridge was due to the Galapagos hot spot activity (GHS). The corresponding pressure‐temperature (P‐T) diagram shows the unknown state during this time (red circle). (b) Previous fluid inclusion studies indicate isobaric heating conditions attributed to an advective heat source in the P‐T diagram. *Brandstätter et al*. [2016] constrained three possibly heating events, two are related to the interaction of the Cocos Nazca spreading center (CNS) and the Galapagos hot spot (one of them is shown), and the third possibility is a younger event, where a small spreading center was active and formed the 2 Ma old Cocos Island (Co. Is.). (c) Present situation at the Middle America Trench, subduction of the Cocos Ridge, and drill Site U1414. Deformation is still active, due to the bending of the plate and seismic rupture at cooler temperatures. Red star represents the epicenter of the 2002 *M*_*w*_ 6.4 Osa earthquake [*Arroyo et al*., 2011].

Brittle deformation resulted in the first vein formation in the basalt, fibrous calcite precipitation in the veins, and the development of microcracks in the phenocrysts due to thermal contraction of the cooling basalt (Figure 10a). A subsequent heating event led to hydrofracturing and vein formation in the sedimentary cover (Figure 10b). Successive cooling was associated with twinning in the vein calcite within the sedimentary rocks. Enhanced differential stresses were required for the onset of subgrain formation and mechanical twinning and are ascribed to the approach of the Cocos Ridge to the Middle America Trench and the related peripheral bending of the Cocos Plate and increase of bending‐related intraplate differential stresses adjacent to the subduction zone (Figure 10c). Bending is also documented by the formation of extensional clay mineralized veins and distinct normal faults throughout the sedimentary sequences of Units I–III at Site 344‐U1414 and Units I–IV at hole 344‐U1381C [*Harris et al*., 2013a]. The biostratigraphic record within the uppermost units at Sites U1414 and U1381 revealed Pleistocene to Holocene sedimentation ages [*Harris et al*., 2013a, 2013b] and therefore provides a time constraint for the postsedimentary deformation observed within these units, which is ascribed to Cocos Plate bending, at a time when the aforementioned sites were already within short distance to the Middle America Trench.

Additionally, seismic activity at the trench and aftershocks at the subducting plate triggered late vein formation [*Dielforder et al*., 2015] (e.g., aragonite veins in the basalt), formation of microcracks in the calcite veins and probably enabled further twinning due to stress release [*Lacombe and Laurent*, 1996] (Figure 10c). The latter would explain the observation of twins overprinting subgrains (Figure 4b). *Arroyo et al*. [2011] showed that the majority of earthquake epicenters are located on the upper Caribbean Plate; however, also, a small number of aftershocks of the 1999 Quepos event occurred at the lower Cocos Plate, WSW of Site 344‐U1414.

Therefore, we suggest that the observed microstructures are the result of different deformation processes, operating just after Cocos Ridge basalt formation and subsequent cooling of the oceanic crust, during the movement of the Cocos Plate toward the Middle America Trench and finally at the trench.

## 7 Conclusion

The deformation history at Site 344‐U1414 is characterized by several tectonic processes that occurred close to the spreading center, during the movement of the Cocos Ridge from the Galapagos hot spot to the convergent margin offshore Costa Rica and at the Middle America Trench. A variation of deformation mechanisms affected the rocks at Site 344‐U1414 at low depths (maximum 470 m bsf), resulting in several deformation structures and low strains. Brittle deformation in the sedimentary rocks and in the basalt, such as vein formation, microcracks in phenocrysts, and crack‐seal fabrics, are ascribed to thermal contraction, hydrofracturing, and seismic activity. Solution transfer processes caused bedding‐parallel stylolites in the sedimentary rocks. Crystal‐plastic deformation of the vein filling minerals restricted in the sedimentary rocks, such as calcite twins and subgrains, implies a change of the environmental conditions enabling the overcome of the CRSS of calcite, in a temperature range between 170°C and 220°C and increased differential stresses. These conditions were reached due to the increase of bending‐related intraplate differential stresses.
